# Identity enactment as a social accomplishment: Shared identity and the provision of mutual support amongst pilgrims undertaking the Hajj

**DOI:** 10.1111/bjso.12796

**Published:** 2024-08-27

**Authors:** Enes Yalcin, Nick Hopkins

**Affiliations:** ^1^ University of Dundee Dundee UK; ^2^ Present address: Akdeniz University Antalya Turkey

**Keywords:** Hajj, identity enactment, pilgrimage, social support, solidarity

## Abstract

Experimental and survey research shows that a common group membership can result in increased levels of social support. Here we complement such research with qualitative data concerning the forms and function of such support. Specifically, we explore the mutual support reported by pilgrims undertaking the Hajj. This requires participants enact a series of identity‐related beliefs and values (including specific rituals) in conditions that are practically and psychologically challenging. Using data obtained through semi‐structured interviews (*N* = 33), we investigate how participants' shared identity facilitated their behavioural enactment of these identity‐defining beliefs and values. We focus on how their shared understanding of their beliefs and values as Hajj pilgrims allowed various forms of support (psychological, material, informational, and behavioural) which helped participants translate their identity‐related ideals into behaviour. Our analysis implies that a shared identity provides a frame of reference with which group members can recognize each other's identity‐related concerns and what they need in order to enact their identity. In turn, it implies that in situations where there are practical and psychological constraints on behaviour, action in terms of one's social identity can be conceptualized as a joint accomplishment in which the mutual support of group members is key.

## BACKGROUND

Social psychology has long been interested in the provision of help, its forms, and the circumstances in which it is given and received (e.g., Cohen & Wills, 1985; Pearce & Amato, 1980). Whilst much attention has focused on the individual and situational factors affecting pro‐social behaviour, contemporary research has been transformed by the social identity approach to group processes (Turner et al., 1987) showing how a shared group membership can motivate the provision of support (Haslam et al., 2012; Levine et al., 2005). In the research reported here, we complement such research through exploring the *forms* and *function* of the mutual support found amongst those who identify in terms of a shared social identity. We show how these different forms of support reflect group members' understandings of their shared identity‐related beliefs and values, and the challenges they all face in translating these into behaviour. Accordingly, our analysis highlights the function of group‐based social support in facilitating group members' abilities to translate these shared beliefs and values into practice (what can be called ‘identity enactment’: Hopkins et al., 2023).

Below, we review previous research concerning social support and the circumstances in which it is provided. In doing so, we consider research concerning social support inspired by the social identity approach to group processes and explain the importance of investigating the relationship between group members' shared beliefs and values, the provision of support, and identity enactment. We then present an analysis of these processes using interview data gathered with participants completing the Hajj (the annual Muslim pilgrimage to Mecca). This five‐day event requires pilgrims act in accordance with identity‐defining beliefs and values (e.g., completing a tight schedule of rituals) in conditions that are physically and psychologically challenging. We investigate the role of mutual support in managing such challenges so that pilgrims are able to enact their shared beliefs and values. Indeed, our analysis implies that successful identity enactment (here, completion of the Hajj and its rituals) should be conceptualized as a joint accomplishment made possible by the mutual support arising from participants' shared understandings of their beliefs and values (and the challenges in translating these into behaviour).

### Social identity and the provision of support

The provision of support takes many forms. Sometimes it involves exchanging tangible resources, with Pearce and Amato (1980) differentiating between help i. rendered in a planned/formal versus spontaneous/informal manner; ii. given directly versus indirectly; and iii. addressing serious versus non‐serious recipient needs. Other typologies include the provision of information (McGuire, 1994), or emotional support (Cohen & Wills, 1985). In turn, Gottlieb (1978) differentiates between (i) emotionally sustaining behaviours (e.g., reflecting understanding and encouragement); (ii) problem‐solving behaviours (including the proffering of suggestions and material aid); (iii) interventions which change the wider environment to mitigate stress; and (iv) the provision of a sense of security associated with knowing that others are available should help be needed. Researchers have also found it useful to differentiate between reactive helping (driven by external circumstances) and internally motivated helping (Spitzmuller & Van Dyne, 2013), and between unsolicited proactive helping, unsolicited reactive helping, and solicited reactive helping (Chou & Stauffer, 2016). Still others focus on the motivations involved, differentiating between support based on egoistic, altruistic, and organizational citizenship concerns (Peloza & Hassay, 2007).

Our understanding of the processes relevant to the provision of this broad range of support has been transformed by research associated with the social identity approach to group behaviour (Turner et al., 1987). As is well known, this approach makes the point that just as people can define themselves in terms of their unique individuality, they can define themselves in terms of their social group memberships, and that when doing so there are a series of psychological transformations which have implications for group members' behaviour (Reicher, 2011). One transformation concerns the nature of the beliefs and values relevant for judging behaviour: individuals no longer act on the basis of personal (and thus idiosyncratic) beliefs and values, but on their understandings of the norms, values, beliefs, and interests associated with their psychologically salient social identity. In turn, this means that one's social identity provides the frame of reference with which scenarios are interpreted and decisions made about how to act (Reicher & Hopkins, 2016). Another transformation concerns the social relations between group members: those experiencing a shared identification feel a greater sense of connection and intimacy (even with strangers) than would otherwise be the case (Neville et al., 2022; Neville & Reicher, 2011). Together, these transformations have profound implications for helping.

First, the sense of connection between those with a shared identification impacts the provision of support such that ingroup members receive more than others (Levine et al., 2005; Wakefield et al., 2011). In turn, group members routinely anticipate the provision of support from their fellow group members should they need it (Haslam et al., 2012), with such anticipated support relieving stress (Haslam et al., 2005; Haslam & Reicher, 2006) and contributing to wellbeing and resilience (Drury et al., 2016; Haslam et al., 2018).

Second, a shared identity allows a shared understanding of group members' behavioural norms and the challenges that group members face in translating them into behaviour. This is well‐illustrated in research in intergroup contexts (e.g., protests) where one group's attempts to enact identity‐relevant beliefs and values are opposed by others (e.g., the police). Such research shows that group members' shared purpose can motivate the provision of support designed to overcome the outgroup's opposition (Drury & Reicher, 2005, 2009). The wider implication is that group members' understanding of their shared beliefs and values allows them to identify their own and others' needs for support if they are to enact their shared beliefs and values.

Third, the likelihood of providing support can be shaped by group members' representation of the beliefs and values associated with their group. Most obviously, some groups' norms may represent helping as an identity‐related virtue and so motivate the provision of support (Wakefield et al., 2022). Moreover, norms can shape the experience of such provision: to the degree, one's social identity‐related beliefs and values promote the provision of support, then acting in a supportive manner can itself be experienced (and valued) as a form of identity enactment (Wakefield et al., 2022).

Our research builds on these observations. Analysing interview data gathered with Hajj pilgrims, we explore the support they reported as relevant to their completion of the Hajj. We argue that their shared identity allowed shared understandings of (i) the significance of being able to enact Hajj‐related beliefs, values, and rituals; (ii) the challenges all faced in doing so; and (iii) the religious virtues of proffering support. We argue that in combination, these identity‐related understandings created a social environment in which pilgrims' mutual support was possible and was important in facilitating group members' identity enactment.

### Pilgrimage: Identity enactment and social support

The ability to enact the beliefs and values associated with one's social identities should not be taken for granted. Although constraints on enactment may be particularly apparent in intergroup encounters (Drury & Reicher, 2005, 2009), they can be found elsewhere. For example, enacting one's religious identity can be constrained by one's everyday obligations (Reicher et al., 2021) and by practical constraints on co‐religionists gathering together for communal celebrations (Hopkins et al., 2023). Indeed, according to Durkheim (1912/1995; Olaveson, 2001), it is precisely because of such everyday constraints that some group memberships (e.g., religious communities) schedule gatherings (e.g., pilgrimage events) that provide group members with opportunities to enact their identity.

However, even here identity enactment can be a challenge. If pilgrimage events have as their *raison d'etre* the performance of collective identities (Durkheim, 1912/1995; Olaveson, 2001), they are also typically physically and psychologically challenging. Sometimes they involve rituals that feature self‐harm (Xygalatas et al., 2013). More often, they involve being in physically challenging environments with limited material resources (Pandey et al., 2014). So too they often feature intense crowding (Alnabulsi & Drury, 2014) and movement across significant distances (Yalcin & Hopkins, 2024). Such challenges are not incidental: hardship is integral to the pilgrimage experience (Preston, 1992), and the successful completion of pilgrimage in the face of hardship is key to demonstrating and affirming one's faith and religious identity (Henrich, 2009; Irons, 2001). Accordingly, as pilgrimage events are by their nature challenging, they are ideal contexts in which to investigate the role of social support in identity enactment.

Survey research at various pilgrimage events shows participants can identify in terms of a shared group membership (Alnabulsi & Drury, 2014; Khan et al., 2015). Moreover, such shared identifications can be consequential for participants' positive experience of the event (Hopkins et al., 2016) and the longer‐term outcomes of participation (Khan et al., 2015, 2016; Tewari et al., 2012). A key factor in such outcomes is group members' expectations of social support from their fellows. This is well illustrated in research at the Hajj showing that a shared identity and the associated expectation of social support from others contributed to a more positive experience of crowding (Alnabulsi & Drury, 2014) and also motivated the giving of support (Alnabulsi et al., 2018). However, we know less about the forms and functions of the support provided in such contexts. In some respects, this is because survey research is less suitable for more open‐ended investigations of the diverse forms of support people provide (and their wider significance).

In order to explore the forms and function of group‐based support in such challenging circumstances, our research draws on interview data gathered with pilgrims undertaking the Hajj. Each year, over two million pilgrims from across the world gather in Mecca (Saudi Arabia) to carry out a series of designated rituals within a tight five‐day schedule (Van Steenbergen, 2016). Participants must wear simple clothing that affirms their equality before god and participate in rituals that include prayer, the casting of stones at pillars symbolizing Satan, and circling the Ka'aba – a construction associated with Abraham (Borujerdi, 2018; Sabiq, 1993). The pilgrimage requires participants to walk long distances from holy site to holy site in extreme heat, in conditions of intense crowding (Alkassas et al., 2021). The challenges involved are multiple: the pilgrimage is physically exhausting and requires resolve, especially as pilgrims can become ill (e.g., through dehydration, infection transmission, etc.) and face crushing. Indeed, such are these challenges that a specialist field of medicine (‘mass gathering medicine’) has emerged (Memish et al., 2012). Other challenges arise as pilgrims can be uncertain as to the precise form of the rituals and how best to ensure their behaviour complies with Islamic injunction (e.g., how pilgrims should ensure that norms concerning male–female contact are followed in a crowded environment: Yalcin & Hopkins, 2024).

As noted earlier, the social identity approach to collective behaviour draws our attention to the significance of a shared identity for participants' understandings of their identity‐related beliefs and values and their sense of social connection with each other. An earlier analysis of interview data gathered with Hajj pilgrims (Yalcin & Hopkins, 2024) shows how these features of a shared identity allow a degree of self‐organization amongst participants (e.g., in co‐ordinating their movement in crowded conditions). The current analysis (using these same data) elaborates our understanding of this self‐organization through exploring the ways in which crowd members scaffold each other's abilities to enact their shared identity's beliefs and values.

In approaching our interview data, we worked with a broadly inclusive conceptualization of support. This range is best conceptualized as comprising forms of ‘solidarity’ in which people are not ignored or judged irrelevant but feature (positively) in other group members' calculations of how they should act (Hopkins et al., 2019). Indeed, with regard to the analysis of collective behaviour (e.g., social movements), Dawson and Verweij (2012) develop the concept of ‘constitutive solidarity’ to describe forms of pro‐social behaviour in which people can ‘see’ what ought to be done on the basis of their shared values, meanings, and identity.

Inevitably, the challenges in identity enactment faced by any group likely reflect the particularities of their shared identity (e.g., the nature of beliefs and values that are shared) and the nature of the physical and psychological environment they inhabit (e.g., the stressors that characterize the context). Accordingly, it is through the detailed investigation of the particularities of a group's identity and the environment (physical and social) in which group members act, that we can begin to investigate the meanings attributed to support and how the provision of support can feature in identity enactment.

## METHOD

### Participants

Interviews were conducted with 33 Muslims in Britain (28 males, 5 females, aged 23–66). Participants' ethnic backgrounds were diverse (7 Pakistani, 3 Bangladeshi, 2 Indian, 7 Turkish, 1 Malaysian, 3 Indonesian, 1 Libyan, 1 Nigerian, 2 Malawian, 1 Egyptian, 1 Gambian, 1 Cypriot, 1 Uzbekistani, 1 Venezuelan, and 1 English). All had participated in the Hajj and were recruited via Islamic Centres, mosques, and Hajj travel agencies. Eleven were interviewed within 3 months of completing the 2018 Hajj and 21 were interviewed within 3 months of completing the 2019 Hajj. One participant (07F) was interviewed 9 months after the 2018 Hajj.

### Interviews

The interviews (semi‐structured) were conducted by the first author (a Turkish Muslim male) in mosques, cafes, homes, and offices (*M* duration = 51 mins.). Questions addressed participants' relations and interactions with other pilgrims during the Hajj, their perception of a shared identity, and how and when it was manifested. In this context, questions addressed the ways in which participants supported each other. Sometimes participants reported supporting others, sometimes they reported being supported. Throughout, participants are represented by a numerical identifier and their gender (M/F). Translations of Arabic religious terms and additional explanations are given in parentheses and a pause/incomplete sentence is signified by ‘…’.

The Research Ethics Committee of the University of Dundee granted approval for the study. Participants gave their informed consent before taking part. No form of compensation was given to the participants. Because the interview data could potentially identify the participants, our data are not publicly available. However, interested parties are welcome to contact the authors to discuss our data and interpretation.

### Analytic procedure

As we wished to develop a model of how social identity processes shaped the exchange of support, and how such support contributed to participants' abilities to enact their identity as Hajj pilgrims, our iterative reading of the data was shaped by the logic to Grounded Theory. This approach allows for both the development of codes and consideration of the ways these relate to each other. Grounded Theory can be approached differently (Chun Tie et al., 2019), with some approaches being more sensitive to the ways in which ‘we *construct* our grounded theories through our past and present involvements and interactions with people, perspectives, and research practices’ (Charmaz, 2006, p.10, original emphasis). It is therefore appropriate to note our reading was shaped by our theoretical commitments concerning the social identity perspective on the psychological significance of shared identity (Neville & Reicher, 2011; Reicher, 2011), group support, (Drury et al., 2005, 2009; Haslam et al., 2012), and the challenges of identity enactment (Reicher et al., 2021). We also drew on the insights from the first author – a Muslim male with experience of undertaking the lesser Muslim pilgrimage (known as *Umrah*) at Mecca. Accordingly, our reading of these data benefitted from both ‘outsider’ analytic categories (e.g., the concepts of shared identity and identity enactment) and ‘insider’ knowledge of Islamic beliefs and the various challenges involved in the Hajj.

We read participants' interview data as revealing something of their subjective experiences of crowd processes at the Hajj. Using the coding terminology of Birks and Mills (2015), the first phase of analysis involved creating a series of initial (‘open’) codes (e.g., ‘a sense of common purpose’, ‘helping behaviour’, ‘the experience of cultural differences between participants’). In the intermediate coding phase, we elaborated higher‐order codes such as ‘factors that could contribute to a sense of shared identity’, ‘factors that could subvert a sense of shared identity’, ‘the consequences of a shared identity for behaviour’, etc. In doing so, we began to more explicitly explore the ways in which the material associated with the various codes related to each other. Put another way, we began to undertake a process of ‘axial coding’ in which ‘data are put back together in new ways after open coding, by making connections between [and within] categories’ (Strauss & Corbin, 1990, p. 96). As our theoretical interests developed, the process of advanced coding (Birks & Mills, 2015) focused on material relevant to the enactment of identity‐related beliefs and values. In turn, we explored on how this was bound up with the experience (or not) of a shared pilgrim identity and the various ways in which support was (or was not) given and received. Specifically, we explored participants' experiences of i. the degree to which they experienced a shared identification with other pilgrims; ii. the ways in which this was consequential for their understandings of their own and others' needs; and iii. how such understandings were manifested in the support given and received by group members. This resulted in a theoretical focus on how such identity‐related understandings and social support facilitated their own and others' identity enactments.

Extracts relevant to these issues were identified and compared with each other. As our reading of these materials progressed, we developed an analytic taxonomy of the different forms of support contributing to participants' abilities to overcome the challenges of the Hajj and enact their identity as Hajj pilgrims. Although this taxonomy emerged through an inductive process of constant comparison, it is appropriate to note that our reading of these data was shaped by our wider theoretical sensitivities (Charmaz, 2006; Mills et al., 2006). Based on our iterative reading of the interview data, we identified four forms of support that functioned to facilitate identity enactment. One (‘psychological support’) referred to the provision (whether intended or not) of motivational encouragement to enact identity‐related practices (e.g., rituals). Another (‘material support’) referred to the sharing of material resources (e.g., space to sleep, food, medicine, etc.) which indirectly facilitated pilgrims' identity enactment through reducing the impact of the physical hardships that characterize the Hajj. A third type (‘informational support’) referred to the provision of religious knowledge that allowed pilgrims to understand more clearly the behaviours (e.g., prayers and rituals) required of them as pilgrims. The fourth type (‘behavioural support’) captured the ways in which pilgrims modified their own behaviour to establish a context in which proper (i.e., religiously appropriate) behaviour was possible. To be more specific, although the three other forms of support (psychological, material, informational) involved behaviour, the categorization of ‘behavioural support’ refers more specifically to the ways in which pilgrims co‐ordinated their identity enactment with others to facilitate behaviour that conformed to Hajj‐related ideals.

As should be apparent, the relationship between the support offered and identity enactment could sometimes be indirect (as when the sharing of medicine reduced the impact of ill‐health on identity enactment). However, it could also be more direct (as when information‐sharing provided clarity on how one should behave).

## ANALYSIS

Our analysis is structured into three sections. First, we explore the ways participants spoke of their relations with other pilgrims and how this reflected their shared identification as Hajj pilgrims. Second, we describe and illustrate our fourfold typology of the support exchanged between pilgrims. Third, we illustrate the ways in which participants construed such support as normative (and thus as being itself a form of identity enactment). When reporting our data, we provide multiple examples (Elliott et al., 1999).

### Shared identity and social relations

When discussing social relations amongst pilgrims, participants routinely reported the significance of their shared identity. One feature of this shared identity was the sense of pilgrims forming a horizontal community in which all were of equal value. For example, one observed:

Extract 1

you're performing a religious duty as a Muslim and everyone around you is a fellow Muslim. At that time when you're in that crowd, you know that there is no difference between you and the guy standing next to you or behind you or left or right or front except he is a brother in Islam and he has got an exactly equal right as you, doesn't matter whether he is a billionaire or a millionaire or he's a doctor or he's a scientist or he's a sweeper. 08M

Another feature of this shared identification was a strong sense of shared purpose:

Extract 2

All of the people coming there has come there to meet The Lord, has come there because of his invitation. They accept his invitation. The goals are the same; the intentions are the same; the rituals are the same. There is no otherness in this diversity. It has oneness there. 12M

Again, what is striking here is the sense of community amongst Hajj pilgrims: despite their diversity, crowd members shared the ‘goals’, ‘intentions’, and ‘rituals’ associated with their common identification as pilgrims coming to ‘meet The Lord’ such that (as the participant put it) there ‘is no otherness in this diversity’.

A further feature of sense of community was the recognition that all faced similar challenges in completing the Hajj. For example, referring to seeing people walking together from site to site, one participant explained:

Extract 3

you just see all the people around you, and you all are just struggling at the same time. It's quite nice to see each other, just kind of acknowledge we're all struggling together but it's fine, we're here for the same reason. 06F

Thus far, we have explored the potential for a shared identity amongst Hajj pilgrims and how it can be manifested in terms of a shared understanding of the challenges all face when pursuing their identity‐prescribed rituals. In the next section, we turn to the different forms of support participants reported and how these were relevant to pilgrims' abilities to enact their (shared) identity‐related beliefs and values.

### The role of support in identity enactment: Form and function

Participants' understanding of the behaviours required of them as Hajj pilgrims allowed a shared understanding of the challenges all faced in fulfilling the obligations associated with the Hajj. In turn, this allowed awareness of the forms of support relevant to their identity enactment. As described above, we identified four forms of such support.

#### Psychological support

Psychological support was particularly relevant in relation to pilgrims' struggling with exhaustion. One explained:

Extract 4

I try to, I mean chat with other people. Some people, some of them they feel, “Oh, I am tired,” and I tried to, you know, to make a joke with them so we feel, I mean strong together, something I give them motivation. I say, “Okay, come on, come on, it's very close, it's very close.” 27M

Another explained such psychological support was mutual:

Extract 5

there's certain issues during Hajj which are quite difficult but when you have good friends you just laugh it off, let it go, you just… Or if one person is feeling down the other would encourage the other person, it's nice to have that group to keep you going because it's like an emotional rollercoaster in Hajj. 38F

This provides a clear sense of the scale of the psychological challenges characterizing the Hajj (‘it's like an emotional rollercoaster’) and the importance of pilgrims taking it in turns to lift the emotions of those ‘feeling down’: in the words of the participant, ‘it's nice to have that group to keep you going’.

Participants also reported that other pilgrims' performance of their identity‐related rituals was psychologically motivating. Thus, referring to the injunction to chant prayers when visiting the holy sites (a practice echoing the example of the Prophet: Saidovna, 2022), one participant explained others' example motivated them to overcome their fatigue:

Extract 6

It's recommended to shout on the way to the Ka'aba and coming back or to the stoning… So, you say it for sometimes and then you get tired and then you're silent and then when you hear the other people are shouting then you are encouraged to go along with them as well. So it was encouraging, nice. 42M

This latter example makes the point that others' provision of psychological support is not necessarily intentionally given. Nonetheless, from the vantage point of the participant, others' identity‐related behaviour was judged as psychologically encouraging and as motivating their own identity‐related practice.

#### Material support

A second form of support involved the sharing of material resources. For example, one participant referring to the challenges involved in spending the night at a particular site, explained:

Extract 7

we gave spaces to people that were really struggling for space because it was so late at night. I mean you know they would return the favour by giving us their food or medication, or what not. And it was just like a nice sense of community and kind of the sense of looking after each other. That's what Hajj should be because Hajj is so difficult, isn't it, the physical and the mental aspect of it. 06F

Here, material resources as diverse as physical space, food, and medication were shared (reciprocally) in such a way that each helped the other cope with ritual‐related hardships. Moreover, it was because the challenges (physical and psychological) of the Hajj were ‘so difficult’ that this ‘nice sense of community’ and ‘sense of looking after each other’ was conceptualized as a key feature of the pilgrimage.

Another form of material support involved physical care for tired bodies. For example, one participant explained how they reciprocated others' provision of food through providing massages:

Extract 8

We share food, we share medicine and when they feel not so well, I mean the body, fatigue or so, tired, and I gave them massage and they treat me to some food, something like that. 33M

Needless to say, providing others with a pain‐relieving massage implies a significant sense of intimacy, trust, and consent which both reflects a shared social identity and publicly communicates it. That is, it makes the sense of community mentioned in Extract 7 a palpable reality.

#### Informational support

A third form of support involved the provision of information concerning Islamic belief and practice (of obvious significance for identity enactment). For example, one participant described how they sought out religiously knowledgeable pilgrims who could facilitate their own ritual performances:

Extract 9

You find a group which are making, reciting *du'ās* (religious supplications). So, you recite with them because I'm not very good in remembering *surahs* (chapters in Qur'an) and *ayahs* (verses in Qur'an). People are there with the group and they're reciting behind a person. He is loudly saying it. So, people can listen and all the group behind him is saying what he's saying, what the group leader is saying. So, you said “ok this is the best one. They're saying good prayers.” You understand them because they're saying verses in Qur'an. 11M

Motivated to enact Islamic injunction appropriately, the participant judged himself as lacking in identity‐related knowledge and sought such information. Moreover, the participant's understanding of what was required led him to identify others with the requisite knowledge such that he could more properly enact his pilgrim identity. Put simply, a shared identity paved the way for group members to judge both their information needs and identify relevant information sources.

Participants not only reported seeking religious guidance from others. They also noted other pilgrims would spontaneously approach them to offer such guidance. Thus, one explained, others would sometimes remind them of their identity‐related obligations. Specifically, the participant observed others would caution them about becoming too involved in everyday (non‐religious) conversation with others:

Extract 10

I met a few people, they came and they said, ‘Salam,’ and they introduced themselves and then they reminded me of Allah and they said, “Prophet Muhammed, he said, you need to engage yourself in more *ibadah* (worship) because people can get carried away by talking worldly things.” So, sometimes the people came and they just gave you a reminder and it was nice. 39M

Analytically, this illustrates the point that a shared identity not only encouraged the seeking of informational support from other group members but could entail others pro‐actively providing information (in this case providing information concerning prophetic injunction). More specifically, others' identification with oneself provided both an understanding of one's identity‐related goals and the sense of connection that motivated them to act on that understanding (volunteering the caution against ‘talking worldly things’). We cannot know if the unprompted provision of such guidance on appropriate pilgrim practice was always welcome. In some contexts, it could be resented. However, here the participant is clear that one's fellows' advice was ‘nice’, presumably because it was experienced as facilitating their focus on the beliefs and values associated with the Hajj (i.e., their identity enactment).

#### Behavioural support

A fourth form of support relevant to pilgrims' abilities to enact their shared beliefs and values involved the co‐ordination of their behaviour. Take for example the challenge of prayer in a crowded environment. Islamic tradition requires that prayers are conducted in a clean environment (Akgül & Karadag, 2016) and prayer mats are used to ensure this requirement is met. Reflecting on such issues, one participant explained that rather than placing their mat lengthwise they placed it in such a way that another could share it:

Extract 11

If I know that someone is beside me and she didn't have a mat, I just put my thing horizontally. Then, she can fit on my mat. 07F

Here, a shared identity involving a shared understanding of identity‐appropriate religious practice allowed the participant to anticipate their neighbour's practical needs and motivated the modification of their own religious practice to facilitate the other's. Analytically speaking, such a behavioural modification of one's own devotional practice constitutes an example of group members co‐ordinating their behaviour such that the behaviour of the one scaffolds the other's abilities to follow Islamic injunction concerning prayer (and so more properly enact their identity‐related beliefs and values).

Other examples of such co‐operation and behavioural co‐ordination included scenarios where participants sought to ensure conformity with Islamic gender‐related norms concerning modesty in conditions where unrelated men and women were in close proximity. One participant reported the challenge of men and women sharing a large tent in these terms:

Extract 12

We saw that was not appropriate. So, we made a folding screen between males and females. You can see the synergy there. You immediately become united and try to find a solution. Everybody is positive. Everybody tried to do their best at that moment. Of course, the elderly or the people who do not want to be annoyed may be reluctant to take action or other things. Or I can say everyone may wish an arrangement according to their own desires. This may lead to a bit of friction between people. We experienced such a slight friction, but we made it up. There was not any problem at all. A kind of discussion occurred to separate the tent into the parts in order to use it most efficiently, but this was peacefully solved. 13M

Here, the interviewee described how a scenario (mixed‐sex sharing of a tent) was evaluated against shared beliefs and judged identity‐inappropriate. Moreover, their shared evaluation motivated collective discussion as to what could, and should, be done. Such discussion was not necessarily straightforward (it involved ‘a slight friction’). However, the unity of purpose in finding a solution allowed the behavioural co‐ordination required to establish the necessary gender‐segregated space that complied with identity‐related norms.

### Support as a form of identity enactment

In this section, we explore participants' representation of their acts of solidarity. As will become clear, pilgrims' acts of solidarity were not simply construed as means to facilitate the enactment of identity‐related beliefs and values. They were also represented as normative and thus could themselves be forms of identity enactment.

The normative nature of solidarity amongst Hajj pilgrims was explained by one participant who emphasized that the Hajj was not a spectacle to be observed, but rather an opportunity to act:

Extract 13

when doing Hajj (it) is not like we watch football, like, no, we watch like festivals or something like that. We go to Hajj for *ibadah* (worship), for spiritual journey, for trying to look after each other from other countries, from other, from different people, different culture, different skin, everything. Not only, you know, praying, not only make *du'ās* (praying) and this. (Excluded) Allah makes us, makes our Hajj, makes our brains like the same way, I mean the same as what we think that we have to be patient, we have to look after each other, it's very beautiful. 27M

Here the contrasts with gatherings where one is a spectator (e.g., football) work to emphasize the importance of Hajj pilgrims' own behaviour with the participant defining the behavioural norms at the Hajj as requiring pilgrims ‘look after each other’. Indeed, the injunction to look after each other was represented as a religious duty on an equal footing with such duties as making one's *du'ās* (praying). Moreover, it is striking that this emphasis on mutual support was attributed to a shared understanding of what is appropriate (which in turn was attributed to Allah who ‘makes our brains like the same way’).

The characterization of acts of solidarity as normative was elaborated upon by others who explained that acts of helping brought religious credit. One explained that Hajj pilgrims' acts of solidarity would be divinely rewarded and that this motivated pilgrims to seek out opportunities to perform as many ‘good deeds’ as possible:

Extract 14

Whenever there is a need, you find people there around you. So, helping … So, people are helping. People are trying to gain good deeds as much as they can by offering whatever they can offer to others. 10M

Moreover, some participants explained that the rewards for such good deeds were not abstract but could be tangible and immediate. For example, referring to the way they modified their behaviour to facilitate others' religious practice, one observed:

Extract 15

I always try to always make space for people if anyone was wanting to pray next to me. I always notice that for some reason during that day or that course of the week, God will bring something to me. I don't know, maybe because we were willing to give a space but we should give a space anyway. I noticed things would happen during that day or that week. God gave something else to me. I don't know how to explain. There is more *barakah* (prosperity) in whatever was happening, and maybe it was a result of just being generous (in terms of giving space) in a mosque. 06F

The normative dimension of pilgrims' solidarity was illustrated by others who represented their opportunity to act in solidarity with others as a blessing from Allah. For example, referring to an occasion in which he supported an elderly man perform his religious rituals, one participant explained that when the latter thanked him, he replied:

Extract 16

I was saying to him, “don't be so grateful to me. If you wish to thank, thank to Allah, thank to Allah not me because God actually made me to be there for you. God made me to be available for you.” So, this is really, “if you wish to thank, thank to Allah, not to me.” I'm really lucky the God actually blessed me. 09M

Such a quote underscores the point that Hajj pilgrims' understandings of their acts of solidarity were shaped by their identity‐related beliefs (with this participant representing their own helping as an opportunity and privilege given by Allah). In turn, just as providing support can be represented as identity‐affirming, so too receiving acts of solidarity could be judged identity‐appropriate. Thus, one participant, referring to ritualized acts of sharing coffee and dates when pilgrims approached the *Haram‐i Sharif* (the Grand Mosque), explained:

Extract 17

I was surprised to see that everyone, they're going to the prayers and coming back, regardless of your status, will take something and eat, and take pride into taking that food and eating. No worry feel(ing) there at that time you can afford it or not afford it, because it's in the name of God and it's in name of Allah. So, everyone is happy to distribute and everyone is happy to take that and eat it. So, that was the amazing thing I noticed, because I've noticed normal people just walking, you know sometimes, if you've been really pathetic you say: “hang on a second, I can afford it, why should that take this free food?” but nobody thinks like that over there. 08M

Whereas in other contexts the receiving of food could be judged inappropriate (eliciting the reaction ‘hang on a second, I can afford it, why should that take this free food?’), the participant reports reinterpreting such behaviour through reference to the beliefs and values associated with their shared identity as Hajj pilgrims. Specifically, this giving and receiving was represented as sharing ‘in the name of God’ which all (regardless of their income and status) should participate in. Put another way, both the giving and receiving of food was judged identity‐appropriate. Indeed, from the vantage point of Islamic belief (which specifies that all undertaking the Hajj are equal members of the *ummah*), the sharing of food regardless of one's financial status embodied and enacted Islamic norms of equality and unity, and it is through such giving and receiving that one's pilgrim identification was affirmed.

## DISCUSSION

As explained earlier, various taxonomies of support (e.g., Cohen & Wills, 1985; Gottlieb, 1978; McGuire, 1994; Pearce & Amato, 1980) have been developed which delineate the diverse forms of support (e.g., emotional vs. practical) that people provide and receive across a variety of contexts (e.g., in everyday contexts vs. emergencies). Our research complements this work through providing a taxonomy of the support provided in a specific social context – one in which people sought to enact the beliefs and values of their shared group membership. To our knowledge, this taxonomy is the first to elaborate on the different forms of support found in a large‐scale psychological group (i.e., a crowd context) from the social identity perspective.

Several features of such a context are distinctive. Most obviously where there is a shared identity, the basis for people's behaviour is transformed and this turns what would otherwise be an aggregate of diverse individuals into a psychological group (Reicher, 2011). This means individuals no longer act on the basis of personal (and thus idiosyncratic) beliefs and values, but on their understandings of the group's beliefs, values and norms. Moreover, a shared identification results in a sense of connection and intimacy (even with strangers: Neville & Reicher, 2011) and motivates the provision of support to those defined as one's fellows (Levine et al., 2005; Wakefield et al., 2011).

Previous analysis of our data highlighted the role of shared identity‐related beliefs and values in negotiating the challenges associated with crowding (Yalcin & Hopkins, 2024). Here, we extend our understanding of the social organization of pilgrims' behaviour to explore the ways in which identity enactment is a collective accomplishment in which mutual support is key. Our analysis emphasizes how participants' shared identity facilitated a shared understanding of the importance of being able to act in particular ways (e.g., appropriate completion of the Hajj rituals) and a shared understanding of the challenges that they faced in their identity enactment. In turn, it emphasizes how the more intimate social relations made possible by a shared identity resulted in participants offering and accepting the support that allowed these challenges to be overcome.

The range of support we explored spanned four dimensions. Sometimes it entailed psychological support (e.g., giving and receiving encouragement). Sometimes, it involved the giving and receiving of material support (e.g., food, medicines). On other occasions, the transaction involved the provision of information (e.g., concerning the conduct of Hajj rituals). Elsewhere, it involved the co‐ordination of behaviour to ensure that Islamic norms were properly enacted (e.g., modifying one's prayer rituals to help another pursue their own prayers appropriately, engaging in group‐based problem‐solving). The circumstances of such support varied: support was given (Extracts 4 and 11) and received (Extract 6), spontaneous (Extract 10) and sought (Extract 9), one‐way (Extract 11) and reciprocal (Extracts 5 and 7), person‐to‐person (Extract 8), and collectively developed (Extract 12). Sometimes the provision of support was unintentional but was nevertheless judged significant for those experiencing it (Extract 6).

Yet, despite this variety, there was commonality: each contributed to participants' abilities to enact their (shared) identity‐related beliefs and values in an environment where it was difficult. Whether this contribution was direct or indirect, our analysis highlights the intimate relationship between the provision of support and identity enactment: without the former, the latter was problematic. Moreover, it is also important to note that the (mutual) provision of support could be represented as normative and thus was itself a form of identity enactment.

Inevitably our research has limitations. Our data arise from retrospective interviews and thus may be subject to memory biases. Moreover, these data may reflect self‐presentational biases, for example, those wishing to present themselves as ‘good’ Hajj pilgrims may over‐emphasize their own helping of their fellow pilgrims. Future research could attempt to circumvent the potential biases associated with retrospective interviews through adopting observational methods at the event itself. Yet, although this would be of value, it is likely that such research would also require interviews (e.g., in order to explore people's understandings of the meaning and hence the significance of any act) and so could also be subject to presentational concerns.

With these caveats in mind, our analysis has several implications. Some concern the Hajj; others are more general. With regard to the former, it is appropriate to note that the Hajj is a significant mass gathering that carries various risks (e.g., infection transmission, dehydration, crushing). Accordingly, understanding how group processes can motivate the mutual provision of support (whether psychological, material, informational, or behavioural) could help mass‐gathering practitioners develop interventions that mitigate the risks and maximize the benefits of participation (Hopkins & Reicher, 2017, 2021). For example, it could help dispel some very negative images of crowd processes that if adopted by the authorities could contribute to further risk (see Drury et al., 2015). Moreover, it can suggest interventions that focus on building participants' sense of shared identity and shaping their understanding of how best to support each other. Take head‐shaving – a religious norm for men at the Hajj. As the sharing of razors can be a vehicle for blood‐borne infection transmission (Rafiq et al., 2009), interventions could explain that offering one's razor to another should not be conceptualized as an act of support that facilitates identity enactment but rather as an act which can do significant harm (Hopkins & Reicher, 2021).

Moving beyond the particularities of the Hajj, there are several wider implications to be drawn. One important observation is that we cannot always assume that identity enactment is easy and research could usefully explore the nature of such challenges. This would require attention to both the particularities of the identity in question (e.g., its identity‐related beliefs and values) and the nature of the contextually relevant challenges group members face in their enactment. Such issues may be identified across a range of social contexts beyond pilgrimage events. Take the challenges faced by female employees who find the performance of their organizational role compromised by males' orientating to them in terms of their other identities, for example, as women and mothers (Rafaeli et al., 1997). Overcoming such hurdles can be challenging, and in some situations, group processes may be especially relevant. Building on the work reported here, research addressing the role of others in scaffolding individuals' identity enactments could explore the degree to which those involved develop a shared identity such that there is: i. a shared understanding of what identity enactment entails in terms of one's behaviour; ii. a shared understanding of the barriers to such enactment; and iii. a sense of mutual social connection such that group members are motivated to help each other in their identity enactment. Addressing such issues would enrich our understanding of how identity enactment is conditional on the availability of social support (Reicher et al., 2021).

With regard to the analysis of helping, several insights are transferable. First, our research speaks to the question of how people can align with others such that they recognize and respond to others' needs (Jensen et al., 2014). Our analysis implies that one route involves shared identity. Others' recognition of one's identifications is psychologically and practically important (Ryan et al., 2023), providing a basis for understanding one's identity‐related concerns and needs. To the extent a shared identity results in mutual recognition between group members, it allows each to attribute to their fellows the same identity‐related concerns and needs they themselves experience (Hopkins et al., 2019). Put differently, group members' shared beliefs and values provide a frame of reference with which they can recognize each other's identity‐related concerns and what could be done to address them (e.g., what they need in order to enact their identity). Such an analysis contributes to the social identity literature's approach to helping (through elaborating on the significance of a shared identification). Second, although the specifics of the support we have explored (e.g., sharing one's prayer mat) are tied to the particular group membership at the heart of our research, our taxonomy is specified at a level of abstraction (psychological, material, informational and behavioural) that has transferability across a range of identities and scenarios. Inevitably, the ways in which these four forms of support are exchanged will depend on the relevant identity and environment. Accordingly, the taxonomy may best be conceptualized as a sensitizing framework alerting us to the diverse ways in which support is exchanged.

Future research may contribute to a more differentiated typology of the support to be found in conditions characterized by a shared identity. However, at this stage the broad message is clear: there is much to be gained through conceptualizing identity enactment as something of a joint accomplishment in which mutual support can be key.

## AUTHOR CONTRIBUTIONS

**Enes Yalcin:** Conceptualization; methodology; data curation; formal analysis; project administration; writing – original draft; investigation; funding acquisition; writing – review and editing. **Nick Hopkins:** Conceptualization; supervision; formal analysis; writing – original draft; writing – review and editing.

## CONFLICT OF INTEREST STATEMENT

The authors confirm that there are no potential conflicts of interest with respect to the research, authorship, and/or publication of this article.

## Data Availability

As the data may allow identification of the interviewee, they are not publicly available. However, interested parties may contact the authors to discuss data‐sharing.
